# Supplementary material for: The Geometry of Financial Institutions -Wasserstein Clustering of Financial Data
Source: Math Financ Econ. 2025 Jul 23;19(4):877–900. doi: 10.1007/s11579-025-00394-2 (PMC12722396; doi:10.1007/s11579-025-00394-2)
Supplement: Supplementary file 1 — (pdf 210 KB) [file 11579_2025_394_MOESM1_ESM.pdf]

Supplementary Material: Further Simulation Experiments

Let us here discuss an extended version of our experiment in Section 7.1 to evaluate our algorithm. In Section 7.1 we evaluated our method in the Euclidean setting, NA k-means, as an imputation method. However, without the imputation step it can also be viewed as a clustering algorithm. In the case of Euclidean data it can be viewed as a clustering algorithm for points with missing values. Thus, we can also compare it to classical k-means after imputing the missing data with more standard methods for imputing missing values as used in 7.1. To recall, the standard methods for imputing missing values we consider are mean imputation, median imputation, multiple imputation, K-nearest neighbor imputation, and imputation through linear regression. However, in the case of clustering Euclidean points with missing data we also consider the k-pod method which was introduced in [7]. These authors consider the same loss function as we do in Example 3.2, i.e. (11), however they propose a different algorithm. To perform the comparison, we use the same simulation from Section 7.1, i.e. sampling from a Gaussian Mixture Model with $k = 5$ clusters in 5 dimensions and different settings of missing values. We use precisely the same simulated data, and for each of those we apply classical k-means to cluster the points after imputing them with a standard imputation method. For our proposed method, i.e. NA k-means, and for the k-pod method, no imputation step is needed as these directly cluster points with missing values. To evaluate the clustering results we use the so-called Rand index, also known as the Rand score, introduced in [27], as well as the adjusted Rand index (c.f. adjusted Rand score), introduced in [16]. They both compare a baseline clustering (in our case, the labels from the normal distribution of the Gaussian Mixture Model to which an observation belongs) to a clustering of the data using an imputation and/or clustering algorithm. The Rand score takes values in $[0, 1]$, with 0 meaning nothing is clustered the same, and 1 meaning the clusterings coincide except for renaming of clusters. In particular, a higher Rand score corresponds to a better clustering method. The adjusted Rand score is similar to the Rand score but adjusted for chance. It takes value between -0.5 and 1 with 0 indicating a random clustering.

In Table 3 we report the corresponding mean Rand scores ± standard errors for the simulations of Section 7.1. In Table 4 there are the corresponding adjusted Rand scores.

Table 3. Rand Scores for Euclidean Simulation With $p = 0.15$.

β^{MCAR}	β^{MAR}	β^{MNAR}	**NA k-means**	**mean imp.**	**median imp.**	**KNN**	**multiple imp.**	**LR**	**k-pod**
1	0	0	0.901 ± 0.008	0.876 ± 0.008	0.864 ± 0.008	0.896 ± 0.008	0.897 ± 0.008	0.892 ± 0.008	0.855 ± 0.008
0	1	0	0.907 ± 0.008	0.874 ± 0.007	0.879 ± 0.007	0.905 ± 0.008	0.894 ± 0.008	0.899 ± 0.008	0.865 ± 0.007
0	0	1	0.861 ± 0.009	0.831 ± 0.007	0.836 ± 0.008	0.857 ± 0.008	0.859 ± 0.008	0.859 ± 0.008	0.838 ± 0.008
0.5	0.5	0	0.897 ± 0.008	0.879 ± 0.007	0.863 ± 0.007	0.902 ± 0.008	0.907 ± 0.008	0.893 ± 0.008	0.866 ± 0.007
0	0.5	0.5	0.884 ± 0.009	0.857 ± 0.007	0.855 ± 0.007	0.879 ± 0.008	0.883 ± 0.008	0.881 ± 0.008	0.856 ± 0.008
0.5	0	0.5	0.882 ± 0.008	0.857 ± 0.007	0.853 ± 0.007	0.877 ± 0.008	0.882 ± 0.008	0.888 ± 0.008	0.85 ± 0.007
0.333	0.333	0.333	0.884 ± 0.009	0.865 ± 0.007	0.864 ± 0.007	0.884 ± 0.008	0.885 ± 0.008	0.879 ± 0.009	0.851 ± 0.007

Table 4. Adjusted Rand Scores for Euclidean Simulation With $p = 0.15$.

β^{MCAR}	β^{MAR}	β^{MNAR}	**NA k-means**	**mean imp.**	**median imp.**	**KNN**	**multiple imp.**	**LR**	**k-pod**
1	0	0	0.768 ± 0.018	0.706 ± 0.016	0.68 ± 0.016	0.756 ± 0.016	0.756 ± 0.017	0.746 ± 0.017	0.661 ± 0.016
0	1	0	0.778 ± 0.017	0.699 ± 0.015	0.709 ± 0.015	0.778 ± 0.017	0.752 ± 0.017	0.763 ± 0.017	0.68 ± 0.016
0	0	1	0.669 ± 0.018	0.593 ± 0.015	0.608 ± 0.017	0.661 ± 0.018	0.665 ± 0.017	0.664 ± 0.017	0.617 ± 0.018
0.5	0.5	0	0.758 ± 0.018	0.71 ± 0.015	0.676 ± 0.015	0.768 ± 0.017	0.78 ± 0.016	0.75 ± 0.017	0.68 ± 0.016
0	0.5	0.5	0.727 ± 0.019	0.656 ± 0.016	0.654 ± 0.016	0.714 ± 0.016	0.724 ± 0.017	0.717 ± 0.017	0.658 ± 0.017
0.5	0	0.5	0.719 ± 0.018	0.656 ± 0.015	0.648 ± 0.015	0.704 ± 0.017	0.718 ± 0.016	0.735 ± 0.017	0.644 ± 0.015
0.333	0.333	0.333	0.727 ± 0.019	0.676 ± 0.015	0.676 ± 0.015	0.726 ± 0.017	0.728 ± 0.017	0.716 ± 0.018	0.644 ± 0.016

We can see that the introduced method NA k-means either performs best or lies within the standard error of the best performing method for each setting of missing values when considering

it solely as clustering algorithm for points with missing data. The naive approaches of mean and median imputation are outperformed. Also, the k-pod method, which uses the same loss function (11) but a different algorithm than proposed here, is outperformed in each setting.

Generalizing our Results. In our simulation study, the amount of missing data is controlled by the parameter p, which represents the share of missing values. In Section 7.1 and the previous paragraph, this parameter was fixed at $p = 0.15$. However, to assess the robustness of our method, we now vary p across different values. In Table 5, Table 6 and Table 7 we present the corresponding results analogous to Table 1, Table 3 and Table 4, respectively, when letting $p \in \{0.1, 0.2, 0.25, 0.3\}$.

Table 5. Gromov Wasserstein Distance for Euclidean Simulation With Varying Share of Missing Values.

β^{MCAR}	β^{MAR}	β^{MNAR}	**NA k-means.**	**NA k-means-m**	**mean imp.**	**median imp.**	**KNN**	**multiple imp.**	**LR**
					$p = 0.1$				
1	0	0	0.447 ± 0.021	0.452 ± 0.02	1.021 ± 0.026	1.021 ± 0.026	0.479 ± 0.019	0.625 ± 0.028	0.618 ± 0.024
0	1	0	0.426 ± 0.018	0.427 ± 0.015	1.012 ± 0.028	1.012 ± 0.028	0.445 ± 0.019	0.617 ± 0.025	0.609 ± 0.023
0	0	1	1.171 ± 0.042	1.189 ± 0.041	1.833 ± 0.038	1.833 ± 0.038	1.335 ± 0.038	1.418 ± 0.042	1.391 ± 0.041
0.5	0.5	0	0.406 ± 0.01	0.453 ± 0.033	1.003 ± 0.024	1.003 ± 0.024	0.446 ± 0.016	0.573 ± 0.017	0.584 ± 0.02
0	0.5	0.5	0.874 ± 0.042	0.923 ± 0.039	1.462 ± 0.028	1.462 ± 0.028	1.013 ± 0.033	1.057 ± 0.029	1.045 ± 0.032
0.5	0	0.5	0.856 ± 0.036	0.876 ± 0.036	1.462 ± 0.026	1.462 ± 0.026	0.989 ± 0.031	1.055 ± 0.031	1.061 ± 0.033
0.333	0.333	0.333	0.764 ± 0.036	0.764 ± 0.03	1.33 ± 0.021	1.33 ± 0.021	0.877 ± 0.032	0.908 ± 0.026	0.914 ± 0.028
					$p = 0.2$				
1	0	0	0.605 ± 0.02	0.626 ± 0.021	1.547 ± 0.03	1.547 ± 0.03	0.822 ± 0.016	0.857 ± 0.024	0.879 ± 0.03
0	1	0	0.67 ± 0.036	0.697 ± 0.031	1.589 ± 0.039	1.589 ± 0.039	0.778 ± 0.023	0.914 ± 0.026	0.907 ± 0.026
0	0	1	1.884 ± 0.055	1.918 ± 0.055	2.757 ± 0.062	2.757 ± 0.062	2.204 ± 0.054	2.068 ± 0.061	2.062 ± 0.061
0.5	0.5	0	0.601 ± 0.032	0.618 ± 0.032	1.502 ± 0.03	1.502 ± 0.03	0.779 ± 0.02	0.851 ± 0.023	0.847 ± 0.023
0	0.5	0.5	1.208 ± 0.036	1.286 ± 0.041	2.201 ± 0.036	2.201 ± 0.036	1.612 ± 0.036	1.515 ± 0.04	1.493 ± 0.038
0.5	0	0.5	1.24 ± 0.043	1.3 ± 0.04	2.206 ± 0.037	2.206 ± 0.037	1.644 ± 0.04	1.513 ± 0.043	1.511 ± 0.043
0.333	0.333	0.333	1.006 ± 0.034	1.057 ± 0.036	2.041 ± 0.034	2.041 ± 0.034	1.396 ± 0.037	1.314 ± 0.041	1.33 ± 0.043
					$p = 0.25$				
1	0	0	0.714 ± 0.025	0.73 ± 0.025	1.783 ± 0.033	1.783 ± 0.033	1.023 ± 0.027	0.977 ± 0.022	0.987 ± 0.023
0	1	0	0.783 ± 0.035	0.818 ± 0.031	1.826 ± 0.044	1.826 ± 0.044	0.893 ± 0.027	1.062 ± 0.028	1.081 ± 0.033
0	0	1	2.279 ± 0.06	2.324 ± 0.061	3.177 ± 0.069	3.177 ± 0.069	2.593 ± 0.059	2.414 ± 0.07	2.409 ± 0.069
0.5	0.5	0	0.695 ± 0.026	0.676 ± 0.021	1.742 ± 0.034	1.742 ± 0.034	0.922 ± 0.026	0.981 ± 0.031	0.994 ± 0.033
0	0.5	0.5	1.481 ± 0.044	1.534 ± 0.045	2.544 ± 0.047	2.544 ± 0.047	1.92 ± 0.045	1.759 ± 0.048	1.744 ± 0.046
0.5	0	0.5	1.397 ± 0.038	1.48 ± 0.043	2.574 ± 0.048	2.574 ± 0.048	1.929 ± 0.041	1.743 ± 0.048	1.741 ± 0.047
0.333	0.333	0.333	1.201 ± 0.035	1.313 ± 0.055	2.343 ± 0.039	2.343 ± 0.039	1.638 ± 0.035	1.501 ± 0.038	1.493 ± 0.039
					$p = 0.3$				
1	0	0	0.758 ± 0.028	0.8 ± 0.024	2.009 ± 0.035	2.009 ± 0.035	1.099 ± 0.018	1.095 ± 0.026	1.085 ± 0.022
0	1	0	0.935 ± 0.032	1.013 ± 0.041	2.071 ± 0.05	2.071 ± 0.05	1.015 ± 0.028	1.256 ± 0.033	1.239 ± 0.033
0	0	1	2.719 ± 0.069	2.78 ± 0.069	3.569 ± 0.073	3.569 ± 0.073	2.988 ± 0.063	2.756 ± 0.074	2.757 ± 0.074
0.5	0.5	0	0.816 ± 0.038	0.819 ± 0.033	1.955 ± 0.039	1.955 ± 0.039	1.059 ± 0.02	1.076 ± 0.025	1.082 ± 0.025
0	0.5	0.5	1.585 ± 0.045	1.635 ± 0.045	2.846 ± 0.051	2.846 ± 0.051	2.105 ± 0.046	1.883 ± 0.045	1.898 ± 0.047
0.5	0	0.5	1.6 ± 0.047	1.673 ± 0.047	2.858 ± 0.051	2.858 ± 0.051	2.147 ± 0.044	1.92 ± 0.05	1.92 ± 0.05
0.333	0.333	0.333	1.351 ± 0.043	1.393 ± 0.04	2.656 ± 0.044	2.656 ± 0.044	1.838 ± 0.038	1.662 ± 0.045	1.647 ± 0.04

From Table 5 we conclude that the proposed method NA k-means outperforms all the other considered methods independent of the parameters needed for the missing value generation, when considering the Gromov Wasserstein distance as evaluation measure.

From Tables 6 and 7 we can see that also w.r.t. the (adjusted) Rand score, when NA k-means is solely viewed as clustering procedure for Euclidean points with missing values, it competes with the best performing imputation + clustering techniques. Notably, k-pod is outperformed consistently.

Table 6. Rand Scores for Euclidean Simulation With Varying Shares of Missing Values.

β^{MCAR}	β^{MAR}	β^{MNAR}	**NA *k*-means**	**mean imp.**	**median imp.**	**KNN**	**multiple imp.**	**LR**	***k*-pod**
					$p = 0.1$				
1	0	0	0.906 ± 0.008	0.887 ± 0.008	0.886 ± 0.008	0.911 ± 0.008	0.897 ± 0.008	0.904 ± 0.008	0.875 ± 0.007
0	1	0	0.902 ± 0.008	0.893 ± 0.007	0.888 ± 0.007	0.903 ± 0.008	0.906 ± 0.007	0.906 ± 0.008	0.881 ± 0.007
0	0	1	0.881 ± 0.009	0.864 ± 0.008	0.858 ± 0.008	0.876 ± 0.008	0.88 ± 0.008	0.881 ± 0.008	0.873 ± 0.007
0.5	0.5	0	0.905 ± 0.008	0.888 ± 0.007	0.883 ± 0.007	0.912 ± 0.008	0.906 ± 0.008	0.901 ± 0.008	0.877 ± 0.007
0	0.5	0.5	0.896 ± 0.009	0.874 ± 0.007	0.875 ± 0.007	0.897 ± 0.008	0.885 ± 0.008	0.895 ± 0.008	0.875 ± 0.008
0.5	0	0.5	0.892 ± 0.009	0.878 ± 0.008	0.869 ± 0.007	0.894 ± 0.008	0.891 ± 0.008	0.898 ± 0.008	0.875 ± 0.008
0.333	0.333	0.333	0.896 ± 0.009	0.876 ± 0.008	0.886 ± 0.007	0.905 ± 0.008	0.897 ± 0.009	0.905 ± 0.008	0.883 ± 0.007
					$p = 0.2$				
1	0	0	0.889 ± 0.009	0.845 ± 0.007	0.842 ± 0.007	0.879 ± 0.008	0.88 ± 0.008	0.878 ± 0.008	0.828 ± 0.006
0	1	0	0.884 ± 0.008	0.862 ± 0.007	0.853 ± 0.007	0.883 ± 0.008	0.888 ± 0.007	0.888 ± 0.008	0.834 ± 0.007
0	0	1	0.837 ± 0.009	0.803 ± 0.008	0.808 ± 0.007	0.821 ± 0.009	0.847 ± 0.008	0.844 ± 0.009	0.796 ± 0.01
0.5	0.5	0	0.898 ± 0.008	0.865 ± 0.007	0.848 ± 0.007	0.895 ± 0.007	0.89 ± 0.008	0.891 ± 0.008	0.835 ± 0.007
0	0.5	0.5	0.857 ± 0.009	0.828 ± 0.008	0.835 ± 0.008	0.853 ± 0.008	0.871 ± 0.009	0.868 ± 0.009	0.821 ± 0.008
0.5	0	0.5	0.87 ± 0.008	0.834 ± 0.007	0.832 ± 0.007	0.859 ± 0.008	0.865 ± 0.007	0.866 ± 0.008	0.826 ± 0.007
0.333	0.333	0.333	0.882 ± 0.008	0.835 ± 0.007	0.841 ± 0.007	0.867 ± 0.008	0.88 ± 0.008	0.879 ± 0.008	0.833 ± 0.007
					$p = 0.25$				
1	0	0	0.875 ± 0.009	0.824 ± 0.007	0.82 ± 0.006	0.85 ± 0.008	0.867 ± 0.008	0.867 ± 0.008	0.802 ± 0.007
0	1	0	0.873 ± 0.008	0.839 ± 0.007	0.836 ± 0.007	0.873 ± 0.008	0.873 ± 0.007	0.874 ± 0.008	0.817 ± 0.008
0	0	1	0.815 ± 0.009	0.793 ± 0.008	0.793 ± 0.009	0.785 ± 0.008	0.821 ± 0.009	0.826 ± 0.009	0.77 ± 0.01
0.5	0.5	0	0.891 ± 0.008	0.845 ± 0.007	0.841 ± 0.007	0.87 ± 0.008	0.88 ± 0.007	0.881 ± 0.008	0.817 ± 0.007
0	0.5	0.5	0.851 ± 0.009	0.805 ± 0.007	0.805 ± 0.007	0.829 ± 0.008	0.854 ± 0.008	0.856 ± 0.008	0.794 ± 0.008
0.5	0	0.5	0.855 ± 0.008	0.81 ± 0.007	0.812 ± 0.007	0.827 ± 0.008	0.855 ± 0.009	0.856 ± 0.008	0.796 ± 0.008
0.333	0.333	0.333	0.862 ± 0.009	0.822 ± 0.007	0.825 ± 0.007	0.843 ± 0.008	0.864 ± 0.008	0.864 ± 0.009	0.806 ± 0.008
					$p = 0.3$				
1	0	0	0.87 ± 0.009	0.803 ± 0.007	0.8 ± 0.006	0.849 ± 0.008	0.856 ± 0.008	0.856 ± 0.008	0.765 ± 0.008
0	1	0	0.864 ± 0.008	0.822 ± 0.007	0.828 ± 0.007	0.863 ± 0.008	0.862 ± 0.007	0.865 ± 0.007	0.787 ± 0.007
0	0	1	0.802 ± 0.009	0.781 ± 0.008	0.777 ± 0.008	0.755 ± 0.007	0.81 ± 0.009	0.806 ± 0.008	0.754 ± 0.009
0.5	0.5	0	0.883 ± 0.008	0.825 ± 0.007	0.828 ± 0.006	0.861 ± 0.007	0.868 ± 0.008	0.871 ± 0.007	0.793 ± 0.006
0	0.5	0.5	0.848 ± 0.009	0.798 ± 0.007	0.789 ± 0.007	0.811 ± 0.007	0.846 ± 0.008	0.844 ± 0.008	0.779 ± 0.008
0.5	0	0.5	0.845 ± 0.008	0.792 ± 0.007	0.798 ± 0.007	0.812 ± 0.007	0.84 ± 0.007	0.847 ± 0.007	0.778 ± 0.008
0.333	0.333	0.333	0.862 ± 0.008	0.803 ± 0.006	0.798 ± 0.007	0.833 ± 0.007	0.853 ± 0.008	0.847 ± 0.007	0.78 ± 0.008

TABLE 7. Adjusted Rand Scores for Euclidean Simulation With Varying Share of Missing Values.

β^{MCAR}	β^{MAR}	β^{MNAR}	**NA *k*-means**	**mean imp.**	**median imp.**	**KNN**	**multiple imp.**	**LR**	***k*-pod**
					$p = 0.1$				
1	0	0	0.779 ± 0.017	0.733 ± 0.017	0.732 ± 0.016	0.791 ± 0.017	0.758 ± 0.016	0.774 ± 0.016	0.704 ± 0.015
0	1	0	0.768 ± 0.017	0.748 ± 0.016	0.734 ± 0.015	0.774 ± 0.017	0.779 ± 0.016	0.778 ± 0.017	0.716 ± 0.016
0	0	1	0.72 ± 0.018	0.676 ± 0.017	0.662 ± 0.016	0.707 ± 0.017	0.717 ± 0.017	0.719 ± 0.018	0.698 ± 0.016
0.5	0.5	0	0.776 ± 0.017	0.734 ± 0.016	0.722 ± 0.015	0.793 ± 0.018	0.779 ± 0.016	0.767 ± 0.017	0.711 ± 0.015
0	0.5	0.5	0.757 ± 0.019	0.699 ± 0.015	0.705 ± 0.016	0.757 ± 0.017	0.732 ± 0.018	0.754 ± 0.017	0.705 ± 0.017
0.5	0	0.5	0.744 ± 0.018	0.712 ± 0.016	0.687 ± 0.015	0.752 ± 0.018	0.743 ± 0.017	0.76 ± 0.018	0.705 ± 0.018
0.333	0.333	0.333	0.755 ± 0.018	0.703 ± 0.016	0.73 ± 0.014	0.776 ± 0.017	0.76 ± 0.018	0.776 ± 0.018	0.724 ± 0.016
					$p = 0.2$				
1	0	0	0.741 ± 0.018	0.629 ± 0.014	0.622 ± 0.014	0.718 ± 0.016	0.72 ± 0.016	0.714 ± 0.017	0.593 ± 0.015
0	1	0	0.728 ± 0.017	0.665 ± 0.015	0.649 ± 0.014	0.723 ± 0.016	0.736 ± 0.015	0.733 ± 0.016	0.608 ± 0.015
0	0	1	0.614 ± 0.019	0.528 ± 0.016	0.537 ± 0.016	0.572 ± 0.018	0.634 ± 0.017	0.629 ± 0.018	0.521 ± 0.02
0.5	0.5	0	0.759 ± 0.017	0.674 ± 0.015	0.634 ± 0.014	0.75 ± 0.015	0.739 ± 0.017	0.742 ± 0.017	0.608 ± 0.016
0	0.5	0.5	0.664 ± 0.018	0.592 ± 0.016	0.605 ± 0.017	0.652 ± 0.016	0.697 ± 0.018	0.691 ± 0.017	0.576 ± 0.018
0.5	0	0.5	0.691 ± 0.017	0.598 ± 0.015	0.596 ± 0.014	0.663 ± 0.016	0.677 ± 0.016	0.682 ± 0.017	0.588 ± 0.016
0.333	0.333	0.333	0.719 ± 0.017	0.6 ± 0.014	0.616 ± 0.015	0.683 ± 0.017	0.714 ± 0.016	0.71 ± 0.016	0.598 ± 0.015
					$p = 0.25$				
1	0	0	0.709 ± 0.018	0.583 ± 0.014	0.574 ± 0.014	0.646 ± 0.016	0.689 ± 0.016	0.688 ± 0.016	0.538 ± 0.015
0	1	0	0.698 ± 0.016	0.607 ± 0.015	0.606 ± 0.015	0.699 ± 0.016	0.695 ± 0.016	0.701 ± 0.016	0.566 ± 0.017
0	0	1	0.558 ± 0.019	0.505 ± 0.016	0.508 ± 0.02	0.488 ± 0.017	0.574 ± 0.018	0.583 ± 0.019	0.466 ± 0.02
0.5	0.5	0	0.742 ± 0.017	0.628 ± 0.015	0.616 ± 0.015	0.695 ± 0.016	0.713 ± 0.016	0.719 ± 0.016	0.567 ± 0.015
0	0.5	0.5	0.645 ± 0.018	0.53 ± 0.015	0.532 ± 0.015	0.59 ± 0.016	0.651 ± 0.018	0.656 ± 0.017	0.506 ± 0.018
0.5	0	0.5	0.656 ± 0.017	0.544 ± 0.015	0.552 ± 0.016	0.587 ± 0.016	0.657 ± 0.018	0.658 ± 0.018	0.524 ± 0.018
0.333	0.333	0.333	0.674 ± 0.018	0.573 ± 0.015	0.579 ± 0.016	0.627 ± 0.016	0.677 ± 0.017	0.678 ± 0.018	0.54 ± 0.017
					$p = 0.3$				
1	0	0	0.695 ± 0.017	0.528 ± 0.013	0.525 ± 0.014	0.641 ± 0.015	0.661 ± 0.014	0.662 ± 0.015	0.462 ± 0.014
0	1	0	0.674 ± 0.016	0.571 ± 0.014	0.588 ± 0.015	0.672 ± 0.016	0.667 ± 0.014	0.674 ± 0.015	0.503 ± 0.016
0	0	1	0.522 ± 0.019	0.474 ± 0.015	0.467 ± 0.018	0.407 ± 0.015	0.541 ± 0.019	0.532 ± 0.018	0.425 ± 0.018
0.5	0.5	0	0.722 ± 0.016	0.577 ± 0.014	0.588 ± 0.015	0.669 ± 0.015	0.687 ± 0.016	0.691 ± 0.015	0.51 ± 0.014
0	0.5	0.5	0.634 ± 0.018	0.507 ± 0.014	0.491 ± 0.015	0.543 ± 0.014	0.629 ± 0.016	0.626 ± 0.017	0.478 ± 0.017
0.5	0	0.5	0.624 ± 0.017	0.492 ± 0.014	0.515 ± 0.017	0.542 ± 0.015	0.609 ± 0.016	0.628 ± 0.017	0.469 ± 0.017
0.333	0.333	0.333	0.667 ± 0.017	0.519 ± 0.013	0.511 ± 0.015	0.594 ± 0.015	0.646 ± 0.016	0.629 ± 0.015	0.474 ± 0.016
